# Retrospective Evaluation of the Efficacy of Total Neoadjuvant Therapy and Chemoradiotherapy Neoadjuvant Treatment in Relation to Surgery in Patients with Rectal Cancer

**DOI:** 10.3390/medicina60040656

**Published:** 2024-04-19

**Authors:** Lucian Dragoș Bratu, Michael Schenker, Puiu Olivian Stovicek, Ramona Adriana Schenker, Alina Maria Mehedințeanu, Tradian Ciprian Berisha, Andreas Donoiu, Stelian Ștefăniță Mogoantă

**Affiliations:** 1 Doctoral School, University of Medicine and Pharmacy of Craiova, 200349 Craiova, Romaniaberishatc.sfn@gmail.com (T.C.B.); donoiuandreas@yahoo.com (A.D.); 2 Sf. Nectarie Oncology Center, 200347 Craiova, Romania; ramona_schenker@yahoo.com (R.A.S.); alina.maria591@gmail.com (A.M.M.); 3 Department of Oncology, University of Medicine and Pharmacy of Craiova, 200349 Craiova, Romania; 4 Department of Pharmacology, Faculty of Nursing, Târgu Jiu Subsidiary, Titu Maiorescu University, 040441 Bucharest, Romania; 5 3rd General Surgery Clinic, Emergency County Hospital, 200642 Craiova, Romania; stelian.mogoanta@umfcv.ro; 6 Department of Surgery, University of Medicine and Pharmacy of Craiova, 200349 Craiova, Romania

**Keywords:** rectal cancer, neoadjuvant therapy, total neoadjuvant therapy, chemoradiotherapy, tumor stage

## Abstract

*Background and Objective*: In the therapeutic strategy of rectal cancer, radiotherapy has consolidated its important position and frequent use in current practice due to its indications as neoadjuvant, adjuvant, definitive, or palliative treatment. In recent years, total neoadjuvant therapy (TNT) has been established as the preferred regimen compared to concurrent neoadjuvant chemoradiotherapy (CRT). In relation to better outcomes, the percentage of patients who achieved pathological complete response (pCR) after neoadjuvant treatment is higher in the case of TNT. This study aimed to analyze the response to TNT compared to neoadjuvant CRT regarding pCR rate and the change in staging after surgical intervention. *Materials and Methods*: We performed a retrospective study on 323 patients with rectal cancer and finally analyzed the data of 201 patients with neoadjuvant treatment, selected based on the inclusion and exclusion criteria. Patients received CRT neoadjuvant therapy or TNT neoadjuvant therapy with FOLFOX or CAPEOX. *Results*: Out of 157 patients who underwent TNT treatment, 19.74% had pathological complete response, whereas in the group with CRT (*n* = 44), those with pCR were 13.64%. After neoadjuvant treatment, the most frequent TNM classifications were ypT2 (40.30%) and ypN0 (79.10%). The statistical analysis of the postoperative disease stage, after neoadjuvant therapy, showed that the most frequent changes were downstaging (71.14%) and complete response (18.41%). Only four patients (1.99%) had an upstaging change. The majority of patients (88.56%) initially presented clinical evidence of nodal involvement whereas only 20.9% of the patients still presented regional disease at the time of surgical intervention. *Conclusions*: By using TNT, a higher rate of stage reduction is obtained compared to the neoadjuvant CRT treatment. The post-neoadjuvant-treatment imagistic evaluation fails to accurately evaluate the response. A better response to TNT was observed in young patients.

## 1. Introduction

Worldwide, colorectal cancer represents a challenge due to its high incidence (third place) and mortality (second place). In our country, colorectal cancer ranks first in terms of incidence (13.541 cases/year) and second in terms of mortality (7.381 deaths/year), according to Globocan 2022 (data for Romania) [1]. With reference to rectal cancer, it represents approximately one third of all patients with colorectal cancer, which maintains its positioning among the most frequently encountered types of cancer in medical practice. The most critical prognosis indicators for rectal cancer are the tumor regression grade following neoadjuvant therapy and the pathological staging [2]. A recent analysis of the Surveillance, Epidemiology, and End Results (SEER) database showed an increase in the incidence of rectal cancer in patients under 50 years of age, but the reason for this increase has not yet been explained [3].

In view of the high incidence and mortality of this type of cancer, there is a major and constant interest in the development and improvement of diagnostic and treatment methods. In recent years, due to intensive research on improving treatments for rectal cancer, the recommendations of international guidelines have been modified relatively frequently; one of them is the introduction of total neoadjuvant therapy (TNT) as a type of therapeutic approach. Although TNT is recognized for its therapeutic benefits, long-term side effects have not been well documented [4]. In the therapeutic strategy of rectal cancer, radiotherapy has consolidated its important position and frequent use in current practice due to its indications as neoadjuvant, adjuvant, definitive, or palliative treatment [5]. Neoadjuvant radiotherapy is performed according to the recommendations of international guidelines using one of the following two fractionation regimens: “short-course” radiotherapy (25 Gy/5 fractions) or “long-course” radiotherapy (50.4 Gy/28 fractions) [6]. Based on the results of the German CAO/ARO/AIO 94 study, neoadjuvant chemoradiotherapy (CRT) is included in international guidelines as a treatment for patients with T3-4N0 or N+ rectal cancer [7]. Related to the choice of concomitant radiosensitization chemotherapy, there has been debate regarding the benefits and risks of using 5-Fluorouracil (5-FU) or capecitabine. The results of the NSABP R-04 study [8] and that of Hofheinz et al. [9] demonstrated the equivalence of the two regimens. Several studies demonstrated better outcome in patients who achieved a better pathological response to neoadjuvant treatment. In a retrospective analysis of 566 patients with pathologic complete response (pCR) after neoadjuvant CRT Capirci reported a 5-year disease-free survival (DFS) of 85% and a 5-year overall survival (OS) of 90% [10]. A meta-analysis of the results of 3015 patients from 14 studies showed an important improvement in local recurrence, disease-free survival (DFS), and overall survival (OS) in the 16% of patients who achieved pathological complete response after neoadjuvant treatment [11]. Phase III clinical trials looked at possible benefits of adding oxaliplatin to preoperative chemotherapy with 5-FU or capecitabine. In the CAO/ARO/AIO-04 study, the addition of oxaliplatin to 5-FU significantly increased the percentage of patients with pathological complete response (17% vs. 13%, *p* = 0.031) without a significant impact on grade 3 toxicity (23% vs. 22%) [12,13].

In recent years, total neoadjuvant therapy (TNT) has been established as preferable to concurrent neoadjuvant CRT [6] due to the better results obtained in the studies that analyzed the results of this type of treatment [14,15,16]. In relation to better outcomes, the percentage of patients who achieved pathological complete response after neoadjuvant treatment is higher in the case of TNT [4,17]. By using TNT, not only a higher pathological complete response rate (pCR), but also a better systemic control of the disease is obtained [15].

It is not clearly established whether it is better to start with chemotherapy, then follow with CRT (induction TNT), or the other way around (consolidation TNT), when following a TNT approach [6]. 

Clinical trials in which neoadjuvant CRT was performed demonstrated better results when surgery was carried out after a time interval of several weeks after the completion of radiation therapy. Data from the Lyon R90-01 study show that the chances of down-staging increase when this period is longer than 2 weeks [18]. Sloothaak et al., in a retrospective analysis of 1593 patients treated with preoperative CRT, showed that an interval of 16 weeks from the completion of radiotherapy to surgery was required to achieve a maximal percentage of 16% of pathological complete response among operated patients [19]. In the past, the recommended interval for performing surgery was 4–6 weeks after the completion of neoadjuvant treatment, but this time interval increased to 6–10 weeks in recent clinical trials [20]. 

Preoperative CRT may have a potential anal sphincter-preserving benefit in low rectal cancers. The results of some prospective analysis carried out by the collective from the Memorial Sloan Kettering Cancer Center showed similar 3-year local control rates for operations with resection margins greater than 2cm, less than 2cm, greater than 1cm, and less than 1cm [21,22]. Similar data have been reported by other investigators [23]. The most recommended chemotherapeutic regimens used in TNT for rectal cancer are CAPEOX (capecitabine plus oxaliplatin) and FOLFOX (5-fluorouracil, oxaliplatin, and leucovorin). The multicenter phase III CONVERT study demonstrated the efficacy and safety of CAPEOX as neoadjuvant chemotherapy for patients with rectal cancer, decreasing the incidence of perioperative distant metastases and preventive ileostomy [24]. The effectiveness of FOLFOX in the unadjuvanted treatment of rectal cancer was highlighted in the phase III study PROSPECT NCT01515787 [25].

Neoadjuvant radiotherapy also has long-term benefits in patients with rectal cancer. Patients who received preoperative radiotherapy had a much lower recurrence rate compared to those operated without neoadjuvant treatment (12% vs. 27%; *p* < 0.001), and also an improved 5-year survival (58% vs. 48%; *p* = 0.004). The significant improvement was maintained at 13 years (38% vs. 30%; *p* = 0.008) [26].

The results of the STAR-01 [27], ACCORD [28,29,30], NSABP R-04 [31], and PETACC-6 [32] studies showed that the combination of oxaliplatin did not bring therapeutic benefits nor did it increase the toxicity of the treatment.

The administration of FOLFOX or CAPEOX as neoadjuvant therapy associated with RT, followed by surgery, reduces the occurrence of metastatic disease, increases the interval of disease-free survival, and has an implicit positive effect on the patient’s prognosis, survival, and quality of life [33]. However, additional data are needed regarding the patients’ evolution over time.

In this context, this study aimed to analyze the response to neoadjuvant CRT compared to total neoadjuvant treatment (TNT) regarding the modification of the staging evaluated by means of the pathological result after the surgical intervention, and particularly the determination of the pathological complete response rate (pCR) after neoadjuvant treatment.

## 2. Materials and Methods

### 2.1. Patient Selection, Inclusion and Exclusion Criteria

We conducted a single-institution, retrospective study of 323 patients with rectal cancer admitted to the Radiotherapy Department of Sf. Nectarie Oncology Center, Craiova, Romania, between July 2020 and June 2023, and we analyzed data of 201 patients with neoadjuvant treatment based on the inclusion and exclusion criteria. Patient data were recorded, including age, sex, the histopathological result of the biopsy and surgical specimen, clinical and pathological TNM (8th edition) staging [34], information on the systemic treatment administered prior to surgery, dose and fractionation used in radiotherapy, the result of the post-treatment imaging examination, and information on whether or not surgery was performed. The primary endpoint of our study was to compare pathologic downstaging rate after neoadjuvant CRT vs. TNT. The secondary endpoint was the correlation between imagistic post-therapeutic evaluation and pathological report findings. 

The change in the pathological staging compared to the initial (clinical) staging was followed in response to the neoadjuvant treatment with the classification of this change in 4 subtypes: “unchanged”—no modification of the stage; “upstaging”—increasing the stage of the disease; “downstaging”—reducing the stage of the disease; and “complete response (pCR)”—no signs of disease. 

For the inclusion of patients in the study and the performance of the statistical analysis, the following specific criteria were established.

Inclusion criteria: age over 18 years; indication for neoadjuvant radiotherapy; the patient’s informed consent to the oncological records regarding treatment and the processing of medical data for research purposes; the patient’s option for surgery after neoadjuvant treatment; the diagnosis of adenocarcinoma based on the histopathological examination on sample biopsy; and concurrent CRT with capecitabine or total neoadjuvant therapy (TNT) with CAPEOX or FOLFOX.

Exclusion criteria: “short-course” neoadjuvant radiotherapy; patients without chemotherapy as neoadjuvant treatment (refusal or contraindications); patients who have not completed the neoadjuvant treatment; patients without imaging investigations after neoadjuvant treatment; or patients who did not undergo surgery after neoadjuvant treatment.

**Figure 1:**
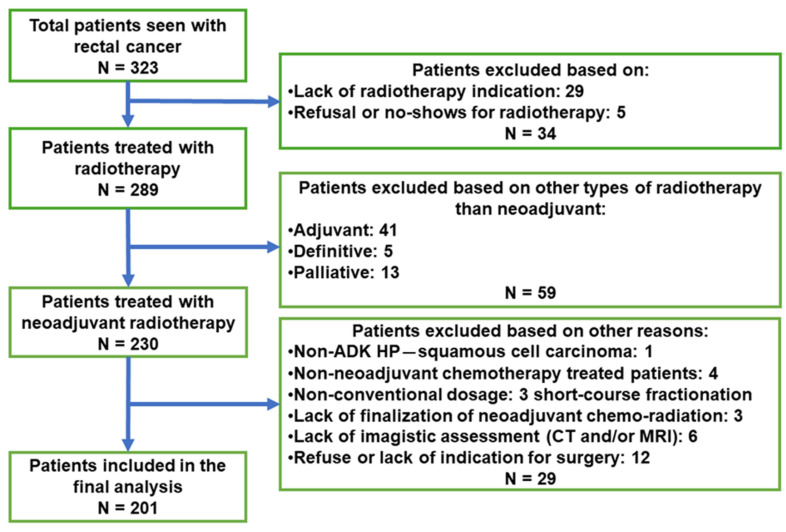
Flow diagram for the inclusion of patients in the study.

From the total number of 323 patients with rectal cancer who presented to radiotherapy consultations, those who did not have an indication for radiotherapy according to NCCN international guidelines and internal hospital protocols were excluded (n = 29) as well those who refused radiotherapy or did not show up for the start of treatment (n = 5), thus leaving a total number of 289 patients with rectal neoplasm who benefited from radiotherapy during the mentioned period. Of the total number of treated patients, 230 benefited from neoadjuvant radiotherapy, while the rest (n = 59) had an indication for adjuvant, palliative, or definitive radiotherapy. From the 230 patients who received neoadjuvant radiotherapy, according to the study criteria, were excluded patients who had another histopathological form of rectal cancer besides adenocarcinoma (n = 1, squamous carcinoma), patients who did not benefit from neoadjuvant chemotherapy (n = 4), patients who received “short-course” neoadjuvant radiotherapy (n = 3), those who did not complete radiotherapy (n = 3), patients who did not show up for imaging control (n = 6), and patients (n = 12) who were not operated upon after radiotherapy (3 patients who refused surgery and 9 patients with unresectable tumors). Finally, the data of 201 treated patients were analyzed (Figure 1).

### 2.2. Indications for Neoadjuvant Therapy

Neoadjuvant treatment was indicated after tumor board analysis based on clinical staging for T1-2 N1-2, T3/T4 cases and at the surgeon’s indication (initially considered unresectable tumors). In patients with oligometastatic disease, the decision regarding inclusion in the study was based on the assessment of the possibility of resection of synchronous hepatic or pulmonary metastases. Thus, 10 patients with stage IVA, who had potentially resectable synchronous hepatic or pulmonary metastases, benefited from neoadjuvant treatment. The association of chemotherapy in the form of TNT or neoadjuvant CRT was proposed by the treating oncologist, and the therapeutic conduct in all analyzed cases was established within the tumor board multidisciplinary oncological team. 

Capecitabine treatment was used as part of CRT and TNT: 825 mg/m^2^, oral administration, two times a day (BID), from Monday to Friday, on days of radiation treatment only, throughout the duration of RT. In the cases where TNT was administered, the chemotherapy was of the CAPEOX type: oxaliplatin 130 mg/m^2^ intravenously day 1 and capecitabine 1000 mg/m^2^ oral administration, BID, for 14 days every 3 weeks; or FOLFOX: oxaliplatin 85 mg/m^2^ intravenously, day 1, leucovorin 400 mg/m^2^ intravenously day 1 and 5-FU 400 mg/m^2^ intravenously bolus on day 1, followed by 1200 mg/m^2^/day × 2 days (total 2400 mg/m^2^ over 46–48 h) continuous infusion. The administration of chemotherapy (CAPEOX or FOLFOX) before CRT was carried out over a period of 12–16 weeks. Patients who performed at least 4 cycles of CAPEOX or 6 cycles of FOLFOX were included in this study. These chemotherapy regimens are recommended by international guidelines [6]. In our institution, the administration of chemotherapy before CRT (induction TNT) was preferred for earlier treatment initiation. In this study, all patients who benefited from TNT underwent chemotherapy before CRT (induction TNT).

### 2.3. Performing Neoadjuvant Radiotherapy

After signing the informed consent, the patients underwent computed tomography scan for the treatment plan. The delineation of target volumes and organs at risk was performed based on the clinical data of the patients and the recommendations of the contouring guidelines [35,36,37,38]. The fractionation used during neoadjuvant radiotherapy was “long-course” 50.4 Gy/28 fractions. The treatment was performed using the Halcyon linear accelerator (manufactured by Varian Pablo Alto, CA, USA). Patients who completed neoadjuvant radiotherapy were recommended to perform imaging control (abdominal-pelvic MRI/CT examination with contrast) at an interval of 4–8 weeks after the completion of treatment. After performing the imaging control, the patients were sent to the surgery service to determine the opportunity of the surgical intervention and its type. Anorectoscopy or anorectal endoscopic ultrasound examinations were performed on surgeon demand for a better surgery planning. The data of the anatomical-pathological examination were collected based on the surgical excision.

### 2.4. Data Analysis

The statistical analysis was performed based on the data obtained from a sample of 201 subjects with rectal cancer. Demographic data and medical characteristics of the study sample were collected as categorical variables and expressed as frequencies and percentages. The comparative analysis of the differences between the 2 groups was carried out by using *t*-tests, chi-square tests, and proportionality tests, because the number of subjects in each group was unequal. The threshold for statistical significance in hypothesis testing was *p* < 0.05. *p*-values were the results of proportionality tests on each scale. The collected data were analyzed using the statistical processing program R v3.5, and the Microsoft Excel application from the Microsoft Office 365 package was used for the descriptive analysis.

## 3. Results

In the analyzed group (N = 201), most patients were men (57.71%) and were aged between 50 and 69 years (52.73%). The average age of the patients was 64.02 years, with standard deviation SD = 10.71 years. The minimum registered age was 34 years and the maximum age was 85 years. The comparative analysis between the ages and the gender of the patients showed that there is no statistically significant difference, suggesting that the age does not differ significantly between the two gender groups (t = 1.289; *p* = 0.199).

From a clinicopathological point of view, most rectal tumors were located in the middle rectum (43.28%) and predominantly had a G2 grade (44.78%). From the point of view of TNM classification, the most frequent classifications were T3 (64.68%), N1b (42.29%), and M0 (95.02%) (Table 1).

After neoadjuvant treatment, the most frequent TNM classifications were ypT2 (40.30%) and ypN0 (79.10%). Imaging evaluation performed before surgery compared with baseline showed that most cases had partial response (PR) (36.81%) and stable disease (SD) (45.27%). The statistical analysis of the postoperative disease stage, after neoadjuvant therapy, showed that the most common stage was stage I (39.80%), compared to zero cases at the initial evaluation. Also, 18.41% of cases had the pCR stage. The most frequent stage changes were downstaging (71.14%) and complete response (18.41%). Only four patients (1.99%) had an upstaging change (Table 2).

The statistical analysis between patient gender and treatment response indicated that there is no statistically significant association between patient gender and imaging response criteria (χ^2^ = 3.502, *p* = 0.321). This suggests that response to treatment, as assessed through imaging, does not depend on patient gender.

The Spearman correlation between patients’ age and treatment response revealed that there is a weak but statistically significant negative correlation between patients’ age and treatment response (correlation coefficient = -0.211, *p* = 0.0027), meaning that younger patients tend to have better responses to treatment than older patients.

The Kruskal–Wallis test to compare the treatment response between different tumor grades (G1, G2, and G3) showed that there are no statistically significant differences between the treatment responses of patients with different tumor grades (H = 1.479, *p* = 0.477). The evaluation of ages in relation to the different stages of the disease did not reveal statistically significant differences (H = 10.455, *p* = 0.0633).

The statistical analysis indicated that there is no statistically significant difference between the tumor grade and the initial stage of the disease (χ^2^ = 16.795, *p* = 0.157) nor between the tumor grade and the postoperative stage of the disease (χ^2^ = 8.889, *p* = 0.838). These results emphasize the complexity of the relationships between tumor characteristics and disease progression.

For categorical variables (gender, location, grade, TNM classification, and initial stage), all *p*-values are greater than 0.05, suggesting no statistically significant differences between treatment groups for these characteristics. This indicates a relatively homogeneous distribution of demographic and clinicopathological characteristics between the groups. For the continuous variable (age), the *p*-value is 0.02085, which is below the significance threshold of 0.05, indicating the existence of significant statistical differences between the treatment groups in terms of patient age.

**Figure 2:**
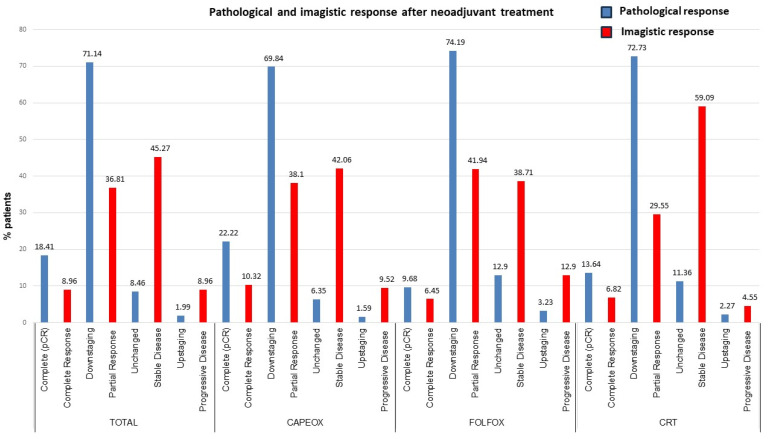
Pathological and imagistic response after neoadjuvant treatment.

From the group of patients treated with CAPEOX, 69.84% of cases presented downstaging and 22.22% complete response. For the arm with FOLFOX, the highest percentages were for downstaging (74.19%) and unchanged (12.9%). In the case of conventional treatment, 72.73% of patients presented downstaging and 13.64% pathological complete response (Figure 2). 

Out of the total number of patients who underwent TNT treatment, 19.74% had a pathological complete response, whereas in the group with CRT, there were 13.64% cases with pCR. The statistical analysis cannot highlight statistically significant differences considering the large difference between the number of patients in each treatment group.

**Figure 3:**
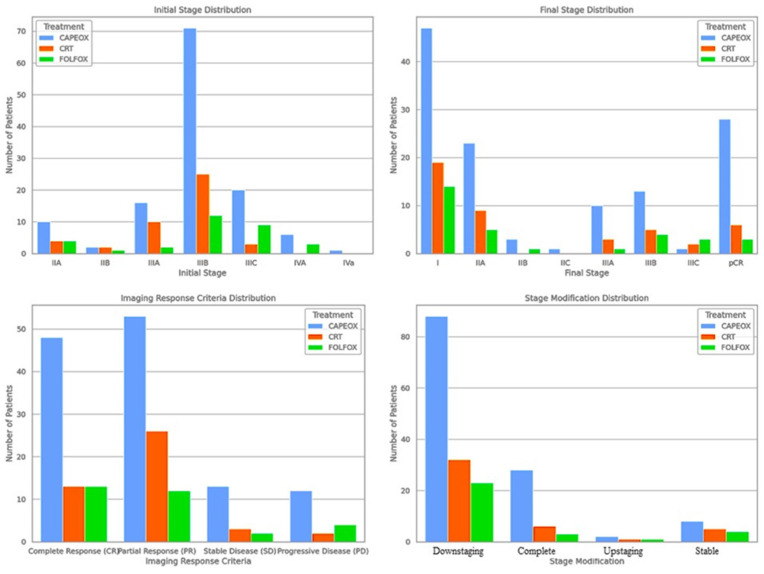
Distribution of patients in the initial stage, the final stage, the imaging response, and the change in the pathological stage, depending on each type of treatment.

For this reason, the statistical analysis was descriptive, to provide a better understanding of the distributions for the initial and final stage, the imaging response, and the change in pathological stage within each treatment group (CAPEOX, FOLFOX, and CRT). From an imaging point of view, in patients treated with CAPEOX the results were as follows: complete response 13 patients (10.32%), progressive disease 12 patients (9.52%), partial response 48 patients (38.10%), and stable disease 53 patients (42.06%). In the case of the group treated with FOLFOX, it was observed that 2 patients (6.45%) had complete response, 4 patients (12.90%) progressive disease, 13 patients (41.94%) partial response, and 12 patients (38.71%) stable disease. In the case of the CRT group, 3 patients had complete response (6.82%), 2 patients progressive disease (4.55%), 13 patients partial response (29.55%), and 26 patients stable disease (59.09%). The descriptive analysis highlighted large differences between imaging and histopathological evaluation, after neoadjuvant treatment, both in the total group and in each treatment arm (Figure 3).

The analysis of the relationship between cancer location and treatment types for 201 patients (87 with median, 75 with lower, and 39 with upper locations) revealed no significant difference in treatment distribution or in the independence between cancer location and treatment choice (*p* = 0.171). Similarly, imaging response and stage changes showed no significant variance across different rectal cancer locations, with imaging response *p*-value closely nearing significance (*p* = 0.087). However, a statistically significant difference was observed in post-treatment tumor classification (ypT) by location (*p* = 0.022), suggesting location may impact treatment response. Despite this, direct comparisons between locations using the Mann–Whitney test did not yield significant differences, likely due to reduced statistical power in individual tests for small sample sizes. No significant differences were found in post-treatment lymph node classification (ypN, *p* = 0.744) or in the final cancer stage (*p* = 0.118), though the latter approached significance.

## 4. Discussion

In the USA, the male–female incidence ratio of rectal cancer is estimated to be 1.39:1, and the median age at diagnosis is in the seventh decade of life [39]. The results of our study are consistent with these epidemiological data, with the male–female ratio being 1.36:1 and the average age being 64.02 years. 

TNT in rectal cancer is a relatively new treatment strategy that involves the administration of a combination of neoadjuvant chemotherapy and radiotherapy before surgery. This approach is used to treat the tumor before surgery, with the aim of improving the chances of success of the surgery, facilitating the surgical removal of the tumor, and reducing the risk of recurrence. After the administration of neoadjuvant treatment, surgery is performed to remove the rest of the tumor and assess the pathological stage of the disease.

Patients who underwent TNT treatment had chemotherapy options with FOLFOX and CAPEOX. This therapeutic combination determined positive results for the patients when evaluating the postoperative stage (complete response 18.41%, downstaging 71.14%). Only 7.64% of patients had unchanged stage and 1.91% had upstaging.

A similar efficacy of TNT was demonstrated in another study in patients who received FOLFOX and chemoradiotherapy (50.4 Gy in 28 fractions) before surgery. Half of the patients received chemotherapy first, and the other half received radiation therapy. Consolidation resulted in better adherence to radiation (97% vs. 91%) but worse adherence to chemotherapy compared with induction (85% vs. 92%). However, the pCR percentage was better (25%) in the consolidation group compared to induction (17%) [40].

Among the patients included in our study, only 11.44% were in stage II of the disease (IIA and IIB), with the majority (88.56%) being diagnosed in advanced stages of the disease (III and IVA). The distribution of postoperative stage after neoadjuvant therapy was substantially changed, with only 20.89% of patients remaining in locally advanced stages (IIIA, IIIB, and IIIC) and 20.9% in stage II (A–C), while a percentage of 39, 8% of the patients were classified as stage I of the disease, and 18.41% of the total analyzed patients obtained a pathological complete response.

It should be noted that in the present study, patients (n = 10) with metastatic disease (stage IVA, potentially resectable synchronous liver or lung metastases) also benefited from neoadjuvant treatment, in which the evaluation of the change in stage took into account only the loco-regional response (ypT and ypN); all these patients showed downstaging after surgery. This fact reinforces the idea that certain patients with oligometastatic disease may benefit from curative treatment, with a possible positive impact on survival [41].

The majority of patients (88.56%) in this analysis initially presented clinical evidence of nodal involvement, and the analysis of the results after the surgical intervention shows that only 20.9% of the patients still presented regional disease.

In this study, differences related to patient gender and parameters such as staging or evolution were not statistically significant. Even if the incidence is higher in men, with an age distribution of 59–60 years, the differences are statistically insignificant correlated with the staging of the cancer at diagnosis and postoperatively. These results correlate with the fact that no gender differences were observed in the diagnosis of more advanced disease and the age-standardized 5-year survival is similar between the sexes [42].

The absence of notable correlations between the tumor grade and the initial disease stage, as well as between the tumor grade and the postoperative stage and treatment response, underscores the complexity of this localized disease. It emphasizes the necessity to explore additional factors, such as specific molecular markers, to better predict the potential response to neoadjuvant treatment.

Older people may have a reduced tolerance to chemotherapy treatments and side effects may be more pronounced. However, it is essential to assess each patient’s situation individually. Some older patients may tolerate treatment well, while others may need dose adjustments or the choice of other therapeutic options. The better response of young patients to neoadjuvant therapy is an additional argument for starting treatment as early as possible from the moment of oncological diagnosis, and thus the application of screening measures for rectal cancer is very important [43].

Data from the literature regarding the pathological complete response rate (pCR) after neoadjuvant treatment in rectal cancer in relation with the age of the patients are contradictory. Some authors present results that show a better response to treatment in the case of young people [44], whereas in other studies the results are worse [45,46]. Possible mechanisms to explain these differences are given by means of molecular and clinical characteristics. Lieu et al. found that in microsatellite stable tumors, TP53 and CTNNB1 alterations were more common in younger patients while APC, KRAS, BRAF, and FAM123B were more commonly altered in older patients. Although rates of alterations in microsatellite genes were similar between young and old patients, when focusing on MSI-H tumors, alterations in APC and KRAS were more common in younger patients, while BRAF alterations were more common in older patients [47]. These differences might partially explain differences in pCR rates [44].

Another possible mechanism to explain this difference in response would be a possible better oxygenation at the tumor level (reduced areas of hypoxia), which leads to an increase in the effectiveness of neoadjuvant radiotherapy in the case of young people.

Considering that patients have a great fear regarding the side effects of chemotherapy and radiotherapy, psychological counseling was necessary. The discussions with the psycho-oncologist facilitated the patient’s communication with the members of the multidisciplinary team, including the clinical pharmacologist [48,49,50,51]. 

In our study, the lack of significant statistical correlations between the treatment response assessed by means of the imaging investigation and that objectivized by the histopathological examination of the surgical excision is not unexpected. Even if they are frequently used in medical practice to assess the response to neoadjuvant treatment, CT and MRI examinations are not reliable to assess the change in the stage of the disease by reducing the size and invasion of the tumor, the disappearance or reduction of regional adenopathy, nor regarding the pathological complete response (pCR). This fact is supported by the results of meta-analyses that assessed this subject [52,53]. Several clinical studies have shown an important reduction in FDG capture when examining PET-CT in patients who responded to neoadjuvant treatment compared to those who did not respond [54]. The current favorable situation in Romania regarding the possibility of performing the PET-CT exam more frequently, due to the emergence of several medical imaging centers that offer this type of examination, could help us in the future to conduct a study on the accuracy of the PET-CT examination concerning the response to neoadjuvant treatment in patients with rectal cancer. 

Since our study is a retrospective one, we must also acknowledge several limitations, starting with the unbalanced arms of neoadjuvant CRT vs. TNT. Also, the short follow-up period did not allow an evaluation of long-term results. Another important issue that we identified while collecting data was the presence of inadequate/incomplete/unstandardized pathologic reports since the patients included in the study addressed different surgical services and, consequently, different pathologic laboratories. The most frequent reporting error was related to the status of margins through the use of a vague statement like “uninvaded margins” without specifying which margins were evaluated. On the other hand, our study “observes” the introduction of TNT approach for rectal cancer in a real-world environment away from the controlled conditions of a trial.

## 5. Conclusions

Neoadjuvant therapy has a major role in downstaging patients with rectal cancer, allowing a better surgical approach. Our results show that by using TNT, a higher rate of the stage reduction is obtained compared to the neoadjuvant CRT treatment, and in particular, a higher percentage of patients obtained pathological complete response (pCR) in the case of TNT versus CRT. The post-neoadjuvant treatment imagistic evaluation fails to accurately evaluate the response since fibrosis or post-radiotherapy lesions might be confounded with neoplastic extensions that do not have a pathologic correspondence on resected specimens. For a more accurate evaluation of the post-therapeutic results, the standardization of pathologic reports of the resected specimen is mandatory. The better response to TNT observed in our study in young patients underscores the need to expand screening limits to younger ages, as these patients may benefit from an early evaluation, diagnosis, initiation of TNT, and surgery.

## Figures and Tables

**Table 1:** Distribution of patients according to demographic and clinicopathological characteristics.

Characteristics		Total Group		CAPEOX		FOLFOX		CRT	
Demographic Characteristics		N	%	N	%	N	%	N	%
Gender	M	116	57.71	75	59.52	17	54.84	24	0
	F	85	42.29	51	40.48	14	45.16	20	100
Age (years)	34–49	22	10.95	18	14.29	2	6.45	2	5
	50–69	106	52.73	66	52.38	19	61.29	10	50
	70+	73	36.32	42	33.33	10	32.26	21	45
Clinicopathological characteristics		N	%	N	%	N	%	N	%
Rectal tumor location	Inferior	75	37.31	50	39.68	8	25.81	17	38.64
	Superior	39	19.41	51	40.48	14	45.16	22	50
	Middle	87	43.28	25	19.84	9	29.03	5	11.36
Tumor differentiation grade	G1	65	32.34	41	32.54	10	32.26	14	31.82
	G2	90	44.78	58	46.03	14	45.16	18	40.91
	G3	46	22.88	27	21.43	7	22.58	12	27.27
T	T1	6	2.98	3	2.38	0	0	3	6.82
	T2	36	17.91	24	19.05	2	6.45	10	22.73
	T3	130	64.68	82	65.08	22	70.97	26	59.09
	T4a	21	10.45	13	10.32	4	12.90	4	9.09
	T4b	8	3.98	4	3.17	3	9.68	1	2.27
N	N0	23	11.44	12	9.52	5	16.13	6	13.64
	N1a	26	12.94	18	14.29	2	6.45	6	13.64
	N1b	85	42.29	52	41.27	11	35.48	22	50
	N2a	46	22.89	31	24.60	7	22.58	8	18.18
	N2b	21	10.44	13	10.32	6	19.35	2	4.55
M	M0	191	95.02	119	94.44	28	90.32	44	100
	M1a	10	4.98	7	5.56	3	9.68	0	0
Stage at diagnosis	IIA	18	8.95	10	7.94	4	12.90	4	9.09
	IIB	5	2.49	2	1.59	1	3.23	2	4.55
	IIIA	28	13.93	16	12.70	2	6.45	10	22.73
	IIIB	108	53.73	71	56.35	12	38.71	25	56.82
	IIIC	32	15.92	20	15.87	9	29.03	3	6.82
	IVA	10	4.98	6	4.76	3	9.68	0	0

**Table 2:** Distribution of patients according to clinical, radiological, and pathological characteristics after radiotherapy.

Clinicopathological Characteristics		N	%
TNT	YES	157	78.11
	NO	44	21.89
ypT	ypT0	33	16.42
	ypT1	21	10.45
	ypT2	81	40.30
	ypT3	52	25.87
	ypT4a	11	5.47
	ypT4b	3	1.49
ypN	ypN0	159	79.10
	ypN1a	20	9.95
	ypN1b	9	4.48
	ypN1c	5	2.49
	ypN2a	6	2.98
	ypN2b	2	1
Postoperative stage (pathologic evaluation)	I	80	39.80
	IIA	37	18.41
	IIB	4	1.99
	IIC	1	0.50
	IIIA	14	6.96
	IIIB	22	10.95
	IIIC	6	2.98
	pCR	37	18.41
Stage change (according to pathologic report) Total lot N = 201	Complete response (pCR)	37	18.41
	Upstaging	4	1.99
	Downstaging	143	71.14
	Unchanged	17	8.46
Stage change TNT CAPEOX N = 126	Complete response (pCR)	28	22.22
	Upstaging	2	1.59
	Downstaging	88	69.84
	Unchanged	8	6.35
Stage change TNT FOLFOX N = 31	Complete response (pCR)	3	9.68
	Upstaging	1	3.23
	Downstaging	23	74.19
	Unchanged	4	12.90
Stage change CRT N = 44	Complete response (pCR)	6	13.64
	Upstaging	1	2.27
	Downstaging	32	72.73
	Unchanged	5	11.36
Imagistic post-neoadjuvant evaluation Total lot N = 201	Complete Response	18	8.96
	Progressive Disease	18	8.96
	Partial Response	74	36.81
	Stable Disease	91	45.27
Imagistic post-neoadjuvant evaluation TNT CAPEOX N = 126	Complete Response	13	10.32
	Progressive Disease	12	9.52
	Partial Response	48	38.10
	Stable Disease	53	42.06
Imagistic post-neoadjuvant evaluation TNT FOLFOX N = 31	Complete Response	2	6.45
	Progressive Disease	4	12.90
	Partial Response	13	41.94
	Stable Disease	12	38.71
Imagistic post-neoadjuvant evaluation CRT N = 44	Complete Response	3	6.82
	Progressive Disease	2	4.55
	Partial Response	13	29.55
	Stable Disease	26	59.09

## Data Availability

Data are contained within the article. All data are available by request to the corresponding authors.
